# Factors influencing postpartum depression in Saudi women: a cross-sectional descriptive study

**DOI:** 10.4069/whn.2024.06.18

**Published:** 2024-06-28

**Authors:** Amira Alshowkan, Emad Shdaifat

**Affiliations:** Community Nursing Department, College of Nursing, Imam Abdulrahman Bin Faisal University, Dammam, Saudi Arabia

**Keywords:** Demography, Depression, Postpartum period, Saudi Arabia

## Abstract

**Purpose:**

This study aimed to investigate the prevalence of postpartum depression (PPD) and stress, as well as factors influencing PPD, among women in Saudi Arabia.

**Methods:**

This study employed a cross-sectional online survey and recruited participants during postpartum visits to the Clinic of Gynecology and Obstetrics in Al-Khobar, Saudi Arabia. Data collection was done using Arabic versions of the Edinburgh Postnatal Depression Scale, Perceived Stress Scale, and a sociodemographics and obstetric history questionnaire. Descriptive and inferential analyses were conducted, including multiple linear regression using a stepwise method.

**Results:**

Data from the 270 participants showed low levels of postpartum depressive symptoms with a mean score of 2.54±4.5 and low levels of perceived stress with a mean score of 2.49±6.2. While 94.4% of the participants reported low levels of stress and PPD, 5.6% reported elevated levels (≥10 for PPD, ≥14 for stress). The stepwise regression analysis showed significant results (*p*<.001), accounting for 34% of the variance in PPD. The factors significantly influencing PPD included the type of family, stress, number of abortions, disease during pregnancy, and family income. Importantly, perceived stress emerged as a factor influencing PPD.

**Conclusion:**

Although the majority of participants exhibited low levels of PPD, about 1 in 18 showed elevated levels. The identification of significant influencing factors highlights the need for targeted interventions to effectively address mental health concerns in postpartum women.

## Introduction

The postpartum phase has been identified as a difficult transition period for women due to physical, psychological, and emotional changes [1]. Significant changes in the mother’s familial and interpersonal relationships lead to the experience of varied emotions of joy, pleasure, sadness, and crying bouts. The inability of mothers to cope with these changes may lead to postpartum stress [2]. Stress has several negative consequences on physical health and general well-being, including sleep disturbances, loss of appetite, and poor lifestyle habits. If postpartum depression (PPD) and stress are not addressed, they may escalate into chronic depression [2-4].

PPD is defined according to the Diagnostic and Statistical Manual as a major depressive episode within 4 weeks after childbirth. It is diagnosed based on the presence of depression symptoms nearly every day, with a significant deviation from the previous routine. Mothers are diagnosed when they show either depression or anhedonia, as well as any five of the following symptoms: sleep disturbance, psychomotor agitation or retardation, feelings of worthlessness, fatigue, suicidal ideation or a suicide attempt, indecisiveness, and loss of appetite. Those symptoms can affect a woman’s life and cause distress and or impairment [5].

PPD has a high prevalence among women worldwide. In Saudi Arabia, the prevalence of PPD among women was reported to range from 45.64% [1] to 52.25% [6]. Several studies in Saudi Arabia have explored the prevalence of PPD [7-10]; however, few have focused on identifying comprehensive influencing factors related to PPD [1,6]. Therefore, the high prevalence of PPD among women in Saudi Arabia compared to Western countries prompted this study to explore factors influencing PPD. This study is expected to make a significant contribution by identifying possible strategies to reduce PPD prevalence among women in Saudi Arabia specifically, and the Middle East in general.

Several factors influencing PPD have been reported in previous studies, including sociocultural, biological, psychological, and psychiatric elements. Psychological stress in women, for example, manifests through marital dissatisfaction, parental stress, fear of labor outcomes, postpartum mood swings, and the burden of childcare [11,12]. Prenatal indicators are also associated with anxiety. For example, in some cultures, there is pressure from parental expectations and fear of the outcomes of labor [13,14].

PPD can arise from depression during pregnancy. A review of 16 studies revealed that women who reported depression or antenatal depression and were taking antidepressants during pregnancy had a higher risk of developing PPD [15,16]. Additionally, a history of personality disorders [17] or somatic disorders during the first 3 months of pregnancy has been identified as a risk factor for PPD [18]. Furthermore, women whose newborns had low birth weights or were female also faced a greater risk of PPD [19]. Moreover, among Arab women, having a cesarean section has been identified as a significant predictor of PPD [1].

In Saudi Arabia, the Ministry of Health provides comprehensive prenatal and postpartum care services. Despite this, reports indicate that 47.9% of women miss one or more appointments, and 15.7% are uncertain about attending their follow-up appointments [20]. This contributes to a lack of awareness about PPD, even among those who have experienced it in previous pregnancies and postpartum periods [21]. Thus, it is crucial to investigate the factors associated with PPD in Al-Khobar, which is located in the eastern region of Saudi Arabia, including stress levels and sociodemographic/obstetric and maternal factors.

This study aimed to examine the prevalence of PPD and stress levels among women in the eastern region of Saudi Arabia, as well as to identify its influencing factors. This research is expected to make a significant contribution since previous studies have reported high levels of PPD. The findings of our study will help identify the factors influencing PPD and will provide implications for practice regarding the early detection and treatment of PPD.

## Methods

**Ethical statement:** Ethical approval was granted by the Institutional Review Board at Imam Abdulrahman Bin Faisal University (IRB-2022-04-435). Informed consent was secured from study participants.

### Study design

This study employed a quantitative cross-sectional design. The study adhered to the STROBE (STrengthening the Reporting of OBservational studies in Epidemiology) reporting guidelines (https://www.strobe-statement.org/).

### Study sample

Participants were recruited from a teaching hospital located in the eastern region of Saudi Arabia during their visits to the Clinic of Gynecology and Obstetrics within 6 weeks postpartum. This study employed convenience sampling.

The inclusion criteria were women within the age range of 18 to 49 years, within 6 weeks postpartum, who currently had a husband. The exclusion criteria were women with a self-reported history of depression and having a newborn with anomalies or born preterm. All types of deliveries were considered, except in cases where the newborn had anomalies or was born preterm. We conducted a power analysis using G*Power software [22] to determine the sample size needed for the linear multiple regression analysis. The input parameters were an effect size (f^2^) of 0.10, alpha (α) of 0.05, power (1–β) of 0.80, and 15 predictors. The analysis showed that a total sample size of 201 was required. Factoring in an approximately 20% dropout rate [23], the target sample size was 242 participants. Eventually, a total of 308 participants were enrolled in the study, with 38 exclusions due to a self-reported history of depression (n=7), having a newborn with anomalies (n=4), preterm birth (n=7), or not having a husband (n=20). Thus, the final analysis included 270 women.

### Measurement

Research data were collected using the following tools:

● PPD: The Arabic version [24,25] of the Edinburgh Postnatal Depression Scale (EPDS) was used to evaluate PPD [26]. The EPDS consists of a 10-item self-reported questionnaire that assesses various areas, including anhedonia, self-blame, anxiety, fear or panic, inability to cope, sleep difficulties, grief, crying, and self-harm. Each item is scored on a range from 0 to 3, indicating the severity of symptoms (possible range, 0–30; with a score of 10 or higher representing depressive symptoms). The Arabic version of the EPDS is widely recognized as a reliable and accurate screening tool for perinatal depression [27]. According to the recommendation to employ a threshold of 10 and greater for depression [28], a score of ≥10 indicates the presence of depressive symptoms. The Cronbach’s alpha coefficient in this study was 0.96, indicating high internal consistency.

● Stress: The Perceived Stress Scale (PSS-10) [29] is a widely used self-reported scale for assessing stress in various groups, including postpartum women. This scale comprises 10 items that inquire about an individual’s feelings and thoughts regarding the level of life stressors experienced over the past month. Responses for each item are recorded on a 5-point Likert scale, ranging from 0 (never) to 4 (very often). A higher total score (possible range, 0–40) indicates higher perceived stress levels. The PSS has been translated into Arabic and has demonstrated satisfactory psychometric properties [25]. To interpret the PSS scores in our study, we applied the following categories according to a prior study [29]: scores ranging from 0 to 13 were classified as low stress, scores from 14 to 26 as moderate stress, and scores from 27 to 40 as high stress. The Cronbach’s alpha coefficient in this study was 0.98.

● Sociodemographics and obstetric history questionnaire: A questionnaire was prepared by the study’s researchers based on previous studies, eliciting data related to sociodemographic characteristics (age, education level, employment status for participant and her husband, Income, type of family, number, and gender of children, and support person) and obstetric history (abortion, type of pregnancy, type of feeding, planned pregnancy, and disease during pregnancy).

### Data collection process

The study team conducted the data collection, identifying potential participants through outpatient clinic nurses. Potential participants were nominated by outpatient clinic nurses and were given invitation letters that contained full information about the study and a barcode and weblink for the study questionnaires. Women who were interested in participating in the study filled out electronic self-reported questionnaires at their convenience within 2 weeks of receiving the invitation letters. All eligible individuals received invitation letters containing comprehensive study information and completed electronic self-reported questionnaires.

### Data analysis

Research data were entered and analyzed using IBM SPSS ver. 22 (IBM Corp., Armonk, NY, USA). Descriptive analyses, including mean, standard deviation (SD), and percentages were employed. The independent-sample t-test and analysis of variance were used to measure the difference in the independent variables by the presence of PPD. In addition, Multiple linear regression utilizing a stepwise method was used to regress PPD on PSS, along with the sociodemographic and obstetric history variables as covariates. The result was considered significant at an alpha level of *p*<0.05.

The Mahalanobis distance method [30] was utilized to identify and eliminate outliers from the dataset. It should be noted that all variables utilized in the analysis, including demographic and maternity factors, were treated as categorical variables. Moreover, the significant sample size of 270 participants strengthens the robustness of the analysis, enabling more reliable parameter estimation and mitigating the potential impact of deviations from normality.

## Results

Table 1 provides a description of the participants’ characteristics. Of the 270 women in the study, most were between the ages of 31 and 40 years (35.2%). The majority of the participants had a bachelor’s degree (91.0%), and were employed (90.7%). The majority (86.2%) of families reported having a monthly income of more than 10,000 Saudi Arabian Riyal (SAR), which is roughly 2,666.2 US dollars and comparable to the national mean income of the study period (32,529.7 SAR gross domestic product per capita for the year 2023, [31]). Additionally, most participants reported having one to two children (55.9%), were members of nuclear families (90.3%), and had never had an abortion (90.3%). Women who exclusively breastfed their newborn represented 67.2% of the participants, followed by combined breastfeeding and bottle-feeding (19.3%). The majority had typical vaginal births (82.4%) and planned pregnancies (86.2%). Only a small percentage of pregnant women (13.8%) reported having an illness during pregnancy. In terms of age, education, employment status, and family factors, the study population included a heterogeneous group of women.

As shown in Table 1, mother’s education was significantly associated with depression levels, with women who had a secondary education exhibiting lower depression levels compared to those with elementary (grades 1–6), and higher than university education. The Tukey honest significant difference (HSD) test demonstrated that the discrepancy in depression levels was significant between elementary and secondary education (*p*=.039) and between secondary and university level education (*p*=.005).

The husband’s employment status was significantly associated with depression levels, with women whose husbands were employed displaying lower depression levels than those whose husbands were unemployed (*p*<.001). Women who had a family income of less than 5,000 SAR (1,333.3 US dollars) demonstrated higher depression levels than those with a family income of 5,000 to 10,000 SAR or more than 10,000 SAR (*p*<.001). The Tukey HSD test revealed that the difference in depression levels was significant between 5,000–10,000 SAR and >10,000 SAR (*p*<.001). Furthermore, women from nuclear families exhibited lower depression levels than those from extended families (*p*<.001).

A noteworthy correlation was found between the history of abortions and levels of depression (*p*<.001). Specifically, women with no prior history of abortion exhibited the lowest average depression scores. Moreover, the application of the Tukey HSD test demonstrated statistical significance in the divergence of depression levels between individuals with no abortions and those with one or two abortions (*p*=.001 and *p*=.015, respectively). Additionally, the type of pregnancy was strongly correlated with levels of depression (*p*<.001). Women who have single pregnancies displayed a significantly lower average depression score in comparison to those who have multiple pregnancies.

The type of newborn feeding was also significantly associated with depression levels, with women who exclusively breastfed their babies displaying lower depression levels than those who formula-fed exclusively or used both feeding methods (*p*<.001). The Tukey HSD test revealed that the difference in depression levels was significant between breastfeeding and both feeding methods (*p*<.001). The type of delivery was also significantly associated with depression levels, with women who had a cesarean section displaying higher depression levels than those who had a normal vaginal delivery (*p*=.012). There was no significant association between planned pregnancy and depression levels, but women who had a disease during pregnancy displayed higher depression levels than those who did not (*p*=.008). The number and gender of child(ren) were not significantly associated with depression levels.

As presented in Table 2, the mean score for the EPDS was 2.54 (SD, 4.5), with 94.4% of participants scoring 10 or less, indicating relatively low levels of depressive symptoms. Similarly, the mean score for the PSS-10 was 2.49 (SD, 6.2), with 94.4% reporting low levels (0–13) of stress, while 5.6% reported moderate levels (14–26), suggesting a relatively low level of perceived stress among the participants. In summary, the descriptive statistics show that, overall, the study participants reported low levels of PPD and perceived stress (Table 2).

The stepwise multiple regression analysis conducted in this study revealed that the model was statistically significant (F=27.41, *p*<.001), with the following predictors explaining 33% of variance in PPD. First, participants from extended families displayed a higher susceptibility to PPD than those from nuclear families (B=1.84, *p*<.001). This emphasizes the significant influence of family dynamics on postpartum mental health. Second, the analysis revealed a strong negative correlation between stress levels and PPD (B=–0.55, *p*<.001). Higher stress levels were associated with lower levels of PPD, underscoring the importance of stress management in mitigating the risk of depressive symptoms. Furthermore, participants with a history of one abortion had higher PPD scores than those with no history of abortions (B=0.48, *p*=.007), suggesting that abortions may have consequences on maternal mental health.

Moreover, the absence of diseases during pregnancy exhibited a correlation with lower levels of PPD (B=–0.30, *p*=.020), thus indicating the significance of maternal health throughout pregnancy. Additionally, higher family income levels (5,000–10,000 SAR) were associated with higher PPD scores (B=0.43, *p*=.007), indicating a complex relationship between socioeconomic status and maternal mental health outcomes (Table 3).

## Discussion

The study aimed to determine the prevalence of PPD and stress among women in Saudi Arabia and to identify factors influencing PPD. The study found that while most women experienced low levels of PPD (using the culturally appropriate cutoff of 10) and stress, a smaller number of women experienced higher levels. These results are consistent with previous studies in Saudi Arabia that used the same measurement tool (EPDS) in Qassim (in the central region) [7], and in Jeddah (in the western region) [8]. Meanwhile, recent studies in different regions of Saudi Arabia have reported a high prevalence of PPD. For example, in Al-Kharj (in the east-central region) the PPD prevalence was 32.8% according to an observational study [9]. In Jazan (in the southwest region) the PPD prevalence was 75.7% using the EPDS [32], and in Riyadh (in the central region) the PPD prevalence was 38.5% using the EPDS [33]. The differences in the prevalence of PPD between the current study and previous studies can be attributed to various factors, including different research designs (observational design), data management (different cutoff points), diagnostic criteria, and geographical variations [7,9,34]. Therefore, it is crucial to consider these factors when comparing the prevalence of PPD at the country level or internationally.

Our study identified perceived stress as a factor influencing PPD, by which higher stress levels of stress were associated with lower levels of PPD. This result is contradictory with previous studies [35-37] as those studies reported intercorrelations of stress, anxiety, and depression during the peripartum period. However, a recent Saudi study [38] reported that 95.7% perceived social support from surrounding people. The protective role of social support against stress is well-documented and is likely linked to lower PPD rates. Additionally, this outcome may be viewed through the lens of women’s insights into stress and their effective use of coping strategies that give women more resilience against depressive symptoms during the postpartum period. Thus, we emphasize the importance of stress management in moderating the risk of depressive symptoms. Therefore, assessments, evaluations, and early interventions should be done to manage women’s stress during the postpartum period.

The current study found that employed husbands and high-income families were associated with lower levels of PPD, aligning with previous research [8,33,39]. One study indicated that the prevalence of PPD among women from high-income families was 4.8 times lower than among those from low-income families [8]. However, other studies have found no correlation between family income and PPD prevalence [7,10]. The difference between this study and other studies conducted in Saudi Arabia lies in the method of income categorization. While this study uses actual income brackets (<5,000, 5,000–10,000, and >10,000 SAR), a study in the central region assessed income based on women’s perception of their income as low, moderate, or high [7]. Another study in the western region categorized income simply as sufficient or insufficient [10]. Consequently, the findings concerning income and PPD should be interpreted with caution due to these variations in data categorization and management. It is important to note that this study was conducted in the eastern region of Saudi Arabia, home to the world’s largest oil fields, which likely contributes to high employment opportunities. This may explain why a significant portion of the study sample (81.2%) reported a high monthly income. Therefore, our results may be influenced by the financial support women receive from their husbands, which could enhance their living conditions and help them manage the physiological and psychological changes during the peripartum period.

This study’s finding that women living in nuclear families had lower levels of PPD aligns with a study from Turkey [40], which reported stronger associations between PPD and lower marital relationship quality in traditional and extended family settings. This contrasts with findings from some studies conducted in China [41] and India [42]. The discrepancy may be attributed to the direct support women in nuclear families receive from their spouses and their fewer social obligations compared to those in extended families. Additionally, the differences in results between this study and others may be influenced by cultural variations. Therefore, we encourage future research to explore the relationship between family type and PPD through cross-cultural studies.

In the current study, cesarean section was identified as a significant factor influencing PPD. This observation aligns with several previous studies [33,43,44]. Conversely, a study from Brazil found no correlation between the mode of delivery and PPD [45], while research from Saudi Arabia revealed that women who had spontaneous vaginal deliveries exhibited higher depression levels than those who underwent cesarean sections [21]. Additionally, women with a single pregnancy showed significantly lower average depression scores. This is supported by a multinational study [46], which reported a higher prevalence of postpartum depressive symptoms among mothers of twins—11.3% compared to 8.3% among those with a single pregnancy. This difference may be attributed to the physical discomfort and increased medical risks associated with multiple pregnancies. Furthermore, women who exclusively breastfed their babies exhibited lower rates of PPD. This finding is consistent with research [47] indicating that exclusive breastfeeding is associated with a 53% reduced risk of PPD. The protective effects of breastfeeding, which promote positive interactions between mother and newborn and increase maternal satisfaction, may explain this association.

This study identified disease during pregnancy as a factor influencing PPD. This finding aligns with research conducted in Vietnam [48], which also recognized the presence of disease during pregnancy as a significant factor in the development of PPD. The negative impact of physical illness on the mental health of pregnant women, coupled with concerns about potential complications for the fetus, can explain this association. Additionally, the study revealed that the number of abortions a woman has experienced influences her risk of developing PPD. This observation is consistent with findings from previous studies conducted in India [49], Turkey [50], and Ethiopia [51]. Specifically, one study [51] reported that women with a history of abortion were about twice as likely to develop PPD compared to those without such a history. This increased risk may be attributed to the emotional distress and deteriorating health following an abortion, which adds psychosocial stress in subsequent pregnancies and, consequently, raises the likelihood of perinatal depression.

The mother’s education was significantly associated with depression levels; women with a secondary education exhibited lower depression levels compared to those with other levels of education. This finding aligns with previous research that associates higher educational levels with a reduced risk of PPD and identifies secondary education as a protective factor against PPD [52]. Specifically, secondary education often leads to vocational training, which typically results in intermediate job positions between the lowest and highest levels of educational attainment.

While this study presents significant findings regarding PPD among women in Saudi Arabia, it is important to acknowledge certain limitations. First, although 15 women were identified with PPD, the sample size was too small to permit detailed analysis. Second, the cross-sectional design of this study only assesses the prevalence of PPD at a single point in time and does not account for changes over time. Additionally, the study sample was recruited solely from one university hospital, limiting the generalizability of the results to all Saudi women in the same region.

In conclusion, this study revealed that while most postpartum Saudi women experienced low levels of depression and stress, only a small proportion reported higher levels of depression. Several factors were identified as influencing PPD, including family type, stress, number of abortions, disease during pregnancy, and family income. The nursing team plays a crucial role in the early detection of stress during the early postpartum period. It is essential that postpartum stress is continuously monitored and assessed. Nursing interventions should prioritize education on stress-reduction techniques and provide guidance on seeking further help if stress levels are unmanageable. A practical approach would involve close monitoring throughout pregnancy and the postpartum period, with referrals to psychiatric clinics when necessary. Additionally, we recommend that future Saudi studies on PPD women explore additional factors not covered in this study, such as social support.

## Figures and Tables

**Table 1.** Characteristics of participants in the postpartum period and their relationship with depression levels (N=270)

Characteristic	Categories	N (%)	Mean (SD)	*p*-value
Age (year)	<21	4 (1.4)	13.5 (0.6)	.438
	21–30	94 (32.4)	2.1 (4.2)	
	31–40	102 (35.2)	2.9 (4.6)	
	>40	90 (31.0)	2.1 (4.4)	
Education	Elementary	2 (0.7)	13.0 (0.0)	.002^†^
	Secondary	24 (8.3)	9.8 (6.4)	
	University	264 (91.0)	1.8 (0.8)	
Employment	Employed	263 (90.7)	1.8 (3.5)	.378
	Unemployed	27 (9.3)	9.2 (7.4)	
Husband’s employment	Employed	269 (92.8)	1.7 (3.4)	<.001
	Unemployed	21 (7.2)	13.3 (2.8)	
Family income (SAR)	<5,000	10 (3.4)	13.4 (0.5)	<.001^‡^
	5,000–10,000	30 (10.3)	8.4 (7.6)	
	>10,000	250 (86.2)	1.4 (2.7)	
Type of family	Nuclear	262 (90.3)	1.3 (2.6)	<.001
	Extended	28 (9.7)	14.0 (1.8)	
Number of child(ren)	1–2	162 (55.9)	2.1 (3.9)	.998
	3–4	82 (28.3)	2.7 (4.5)	
	5–6	21 (7.2)	2.3 (4.7)	
	>6	25 (8.6)	5.1 (7.1)	
Gender of child(ren)	Girls	69 (23.8)	5.1 (1.0)	.227
	Boys	29 (10.0)	2.4 (5.1)	
	Both	192 (66.2)	1.5 (2.0)	
Number of abortion(s)	0	262 (90.3)	1.8 (3.4)	<.001^§^
	1	23 (7.9)	8.7 (1.2)	
	2	2 (0.7)	11.5 (1.6)	
	4	1 (0.3)	13.0 (0.1)	
	6	2 (0.7)	13.0 (0.2)	
Type of pregnancy	Single	262 (90.3)	1.6 (0.8)	<.001
	Multiple	22 (7.6)	14.1 (1.1)	
Type of newborn feeding	Breast	195 (67.2)	1.5 (0.8)	<.001^ǁ^
	Bottle	39 (13.4)	6.5 (0.9)	
	Both	56 (19.3)	5.3 (0.9)	
Type of delivery	CS	51 (17.6)	8.0 (1.0)	.012
	NVD	239 (82.4)	1.3 (0.8)	
Planned pregnancy	No	40 (13.8)	7.2 (1.1)	.807
	Yes	250 (86.2)	1.8 (0.8)	
Disease during pregnancy	Yes	33 (13.8)	6.0 (1.3)	.008
	No	257 (88.6)	2.1 (0.8)	

CS: Cesarean section; HSD: honest significant difference; NVD: natural vaginal delivery; SAR: Saudi Arabian riyal. 5,000 SAR is equivalent to 1,333.3 US dollars (2023).

† Tukey HSD test: elementary vs. secondary (*p*=.039), secondary vs. university (*p*=.005).

‡ Tukey HSD test: 5,000–10,000 vs. >10,000 (*p*<.001).

§ Tukey HSD test: 0 vs. 1 (*p*=.001), 0 vs. 2 (*p*=.015).

ǁ Tukey HSD test: breast vs. both (*p*<.001).

**Table 2.** Descriptive statistics and distribution of postpartum depression and stress scores among participants (n=270)

Variable	Minimum	Maximum	Mean (SD)	Categories	N (%)
Postpartum depression	0	24	2.54 (4.5)	No (<10)	255 (94.4)
				Yes (≥10)	15 (5.6)
Stress	0	23	2.49 (6.2)	Low (0–13)	255 (94.4)
				Moderate (14–26)	15 (5.6)

**Table 3.** Factors influencing postpartum depression (n=270)

Variable	B	SE	β	*p*.	R^2^	F	Adjusted R^2^
(Constant)	–0.15	0.12		.204	0.34	27.41	0.33
Type of family (ref: nuclear)							
Extended	1.84	0.25	0.42	<.001			
Stress	–0.55	0.06	–0.56	<.001			
Number of abortion(s) (ref: 0)							
1	0.48	0.18	0.15	.007			
Disease during pregnancy (ref: yes)							
No	–0.30	0.13	–0.12	.020			
Family income (ref: <5,000 SAR)							
5,000–10,000	0.43	0.15	0.15	.007			

ref, Reference; SAR, Saudi Arabian riyal. 5,000 SAR is equivalent to 1333.3 US dollars (2023)
